# Tetra­aqua­bis­[2-(2-nitro­phen­yl)acetato-κ*O*]cobalt(II)

**DOI:** 10.1107/S2056989015002467

**Published:** 2015-02-11

**Authors:** Muhammad Danish, Muhammad Nawaz Tahir, Sana Iftikhar, Muhammad Asam Raza, Muhammad Ashfaq

**Affiliations:** a Department of Chemistry, Institute of Chemical and Biological Sciences, University of Gujrat, Gujrat 50700, Pakistan; b Department of Physics, University of Sargodha, Sargodha, Punjab, Pakistan

**Keywords:** crystal structure, cobalt(II) complex, hydrogen bonding

## Abstract

The mol­ecule of the title compound, [Co(C_8_H_6_NO_4_)_2_(H_2_O)_4_], is centrosymmetric. It is a cobalt(II) complex, bearing two (2-nitro­phen­yl)acetate and four aqua ligands. The coordination around the Co^II^ atom is distorted octa­hedral, defined by four O atoms of water mol­ecules in the equatorial plane and by two carboxyl­ate O atoms at axial positions. The dihedral angles between the benzene ring and the acetate and nitro groups are 61.90 (10) and 19.21 (11)°, respectively. The water mol­ecules form O—H⋯O hydrogen bonds with the nitro and carboxyl­ate groups, leading to a layered structural arrangement parallel to (001).

## Related literature   

The title compound is structurally related to tetra­aqua­bis(acetato-κ*O*)cobalt(II) (Sobolev *et al.*, 2003 ▸), tetra­aqua­bis­(2-nitro­phen­oxy­ethano­ato-κ*O*)cobalt(II) dihydrate (Kennard *et al.*, 1985 ▸), tetra­aqua­bis­((2,4-di­chloro­phen­oxy)acetato-κ*O*)cobalt(II) dihydrate (Tan *et al.*, 2011 ▸), tetra­aqua­bis­[2-(6-amino-9*H*-purin-9-yl)acetato-κ*O*]cobalt(II) (Mishra *et al.*, 2011 ▸) and tetra­aqua­bis­(3,5-di­nitro­benzoato-κ*O*)cobalt(II) tetra­hydrate (Tahir *et al.*, 1996 ▸).
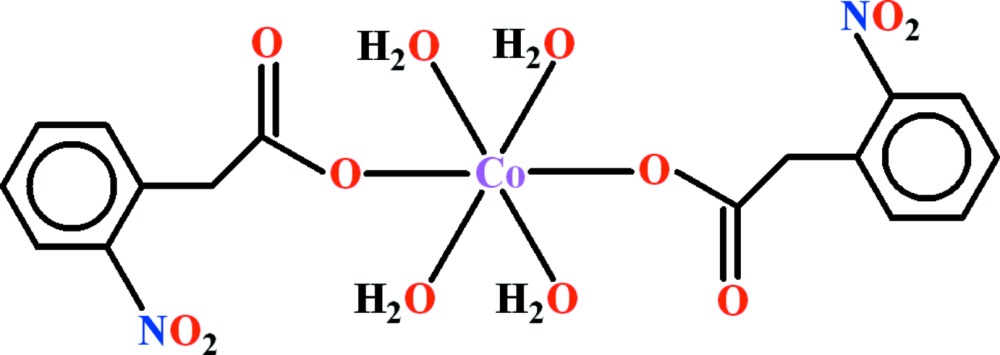


## Experimental   

### Crystal data  

[Co(C_8_H_6_NO_4_)_2_(H_2_O)_4_]
*M* *_r_* = 491.27
Monoclinic, 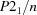

*a* = 5.4431 (3) Å
*b* = 6.4313 (4) Å
*c* = 28.5697 (15) Å
β = 92.762 (2)°
*V* = 998.96 (10) Å^3^
*Z* = 2
Mo *K*α radiation
μ = 0.93 mm^−1^
*T* = 296 K
0.32 × 0.24 × 0.20 mm

### Data collection  

Bruker Kappa APEXII CCD diffractometer
Absorption correction: multi-scan (*SADABS*; Bruker, 2007 ▸) *T*_min_ = 0.758, *T*_max_ = 0.835
8913 measured reflections
2156 independent reflections
1937 reflections with *I* > 2σ(*I*)
*R*_int_ = 0.026

### Refinement  

*R*[*F* ^2^ > 2σ(*F* ^2^)] = 0.028
*wR*(*F* ^2^) = 0.070
*S* = 1.04
2156 reflections
154 parameters
H atoms treated by a mixture of independent and constrained refinement
Δρ_max_ = 0.27 e Å^−3^
Δρ_min_ = −0.28 e Å^−3^

### 

Data collection: *APEX2* (Bruker, 2007 ▸); cell refinement: *SAINT* (Bruker, 2007 ▸); data reduction: *SAINT*; program(s) used to solve structure: *SHELXS97* (Sheldrick, 2008 ▸); program(s) used to refine structure: *SHELXL97* (Sheldrick, 2008 ▸); molecular graphics: *ORTEP-3 for Windows* (Farrugia, 2012 ▸) and *PLATON* (Spek, 2009 ▸); software used to prepare material for publication: *WinGX* (Farrugia, 2012 ▸) and *PLATON*.

## Supplementary Material

Crystal structure: contains datablock(s) global, I. DOI: 10.1107/S2056989015002467/wm5121sup1.cif

Structure factors: contains datablock(s) I. DOI: 10.1107/S2056989015002467/wm5121Isup2.hkl

**Fig. 1.**
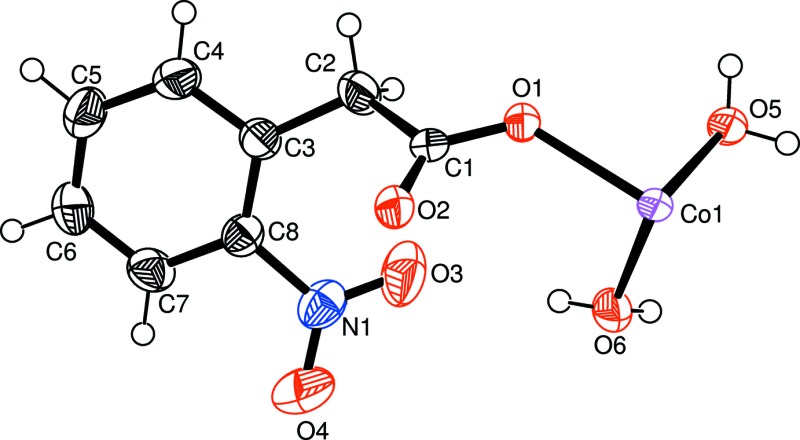
View of the asymmetric unit of title compound with the atom-numbering scheme. Displacement ellipsoids are drawn at the 50% probability level. H-atoms are shown as small circles of arbitrary radius.

. DOI: 10.1107/S2056989015002467/wm5121fig1.tif
View of the asymmetric unit of title compound with the atom-numbering scheme. Displacement ellipsoids are drawn at the 50% probability level. H-atoms are shown as small circles of arbitrary radius.

**Fig. 2.**
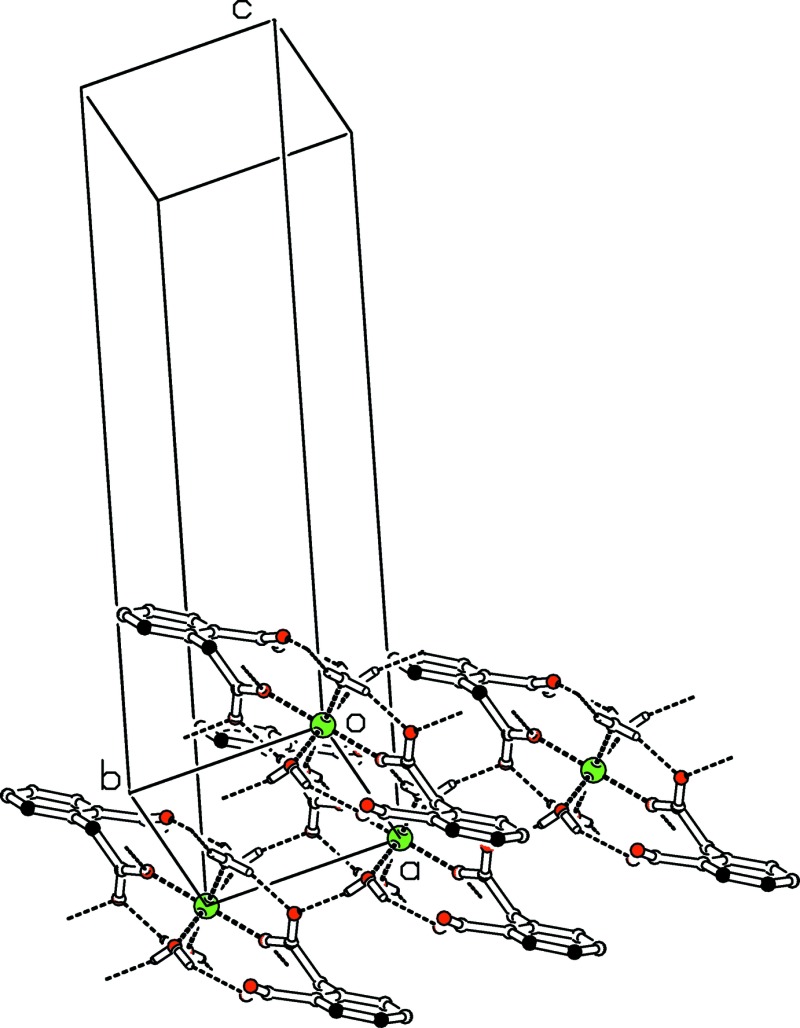
A partial packing diagram of the title compound showing the layered organisation of molecules held together by O—H···O interactions (dashed lines). H atoms not involved in hydrogen bonding are omitted for clarity.

. DOI: 10.1107/S2056989015002467/wm5121fig2.tif
A partial packing diagram of the title compound showing the layered organisation of mol­ecules held together by O—H⋯O inter­actions (dashed lines). H atoms not involved in hydrogen bonding are omitted for clarity.

CCDC reference: 1047494

Additional supporting information:  crystallographic information; 3D view; checkCIF report

## Figures and Tables

**Table 1:** Hydrogen-bond geometry (, )

*D*H*A*	*D*H	H*A*	*D* *A*	*D*H*A*
O5H5*A*O1^i^	0.82(2)	2.00(2)	2.7984(16)	166(2)
O5H5*B*O2^ii^	0.82(2)	1.89(2)	2.6797(17)	161(2)
O6H6*A*O2^iii^	0.78(2)	1.93(2)	2.6979(17)	169(2)
O6H6*B*O3	0.81(2)	2.22(2)	2.988(2)	159(2)

Symmetry codes: (i) 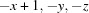; (ii) 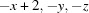; (iii) 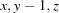.

## supplementary crystallographic information

### S1. Experimental

The sodium salt of (2-nitrophenyl)acetic acid was prepared by mixing aqueous solutions of (2-nitrophenyl)acetic acid and Na(HCO_3_) in the molar ratio 1:1. To this solution a 0.5 molar equivalent of cobalt(II) acetate was added and the mixture refluxed for 4 h. The resulting solution was evaporated for crystal growth. Light-orange prisms suitable for X-ray data collection were obtained after one week.

### S2. Refinement

The coordinates of water H-atoms were refined freely, with *U*_iso_(H) = 1.2*U*_eq_(O). The other H-atoms were positioned geometrically (C—H = 0.93—0.97 Å) and refined as riding with *U*_iso_(H) = 1.2*U*_eq_(C).

### Crystal data

**Table:**

[Co(C_8_H_6_NO_4_)_2_(H_2_O)_4_]	*F*(000) = 506
*M**_r_* = 491.27	*D*_x_ = 1.633 Mg m^−^^3^
Monoclinic, *P*2_1_/*n*	Mo *K*α radiation, λ = 0.71073 Å
*a* = 5.4431 (3) Å	Cell parameters from 2154 reflections
*b* = 6.4313 (4) Å	θ = 2.9–27.0°
*c* = 28.5697 (15) Å	µ = 0.93 mm^−^^1^
β = 92.762 (2)°	*T* = 296 K
*V* = 998.96 (10) Å^3^	Prism, light-orange
*Z* = 2	0.32 × 0.24 × 0.20 mm

### Data collection

**Table:**

Bruker Kappa APEXII CCD diffractometer	2156 independent reflections
Radiation source: fine-focus sealed tube	1937 reflections with *I* > 2σ(*I*)
Graphite monochromator	*R*_int_ = 0.026
Detector resolution: 7.80 pixels mm^-1^	θ_max_ = 27.0°, θ_min_ = 2.9°
ω scans	*h* = −6→5
Absorption correction: multi-scan (*SADABS*; Bruker, 2007)	*k* = −8→8
*T*_min_ = 0.758, *T*_max_ = 0.835	*l* = −36→36
8913 measured reflections	

### Refinement

**Table:**

Refinement on *F*^2^	Primary atom site location: structure-invariant direct methods
Least-squares matrix: full	Secondary atom site location: difference Fourier map
*R*[*F*^2^ > 2σ(*F*^2^)] = 0.028	Hydrogen site location: inferred from neighbouring sites
*wR*(*F*^2^) = 0.070	H atoms treated by a mixture of independent and constrained refinement
*S* = 1.04	*w* = 1/[σ^2^(*F*_o_^2^) + (0.0339*P*)^2^ + 0.3677*P*] where *P* = (*F*_o_^2^ + 2*F*_c_^2^)/3
2156 reflections	(Δ/σ)_max_ < 0.001
154 parameters	Δρ_max_ = 0.27 e Å^−^^3^
0 restraints	Δρ_min_ = −0.28 e Å^−^^3^

### Special details

**Table:**

Geometry. All e.s.d.'s (except the e.s.d. in the dihedral angle between two l.s. planes) are estimated using the full covariance matrix. The cell e.s.d.'s are taken into account individually in the estimation of e.s.d.'s in distances, angles and torsion angles; correlations between e.s.d.'s in cell parameters are only used when they are defined by crystal symmetry. An approximate (isotropic) treatment of cell e.s.d.'s is used for estimating e.s.d.'s involving l.s. planes.
Refinement. Refinement of *F*^2^ against ALL reflections. The weighted *R*-factor *wR* and goodness of fit *S* are based on *F*^2^, conventional *R*-factors *R* are based on *F*, with *F* set to zero for negative *F*^2^. The threshold expression of *F*^2^ > σ(*F*^2^) is used only for calculating *R*-factors(gt) *etc*. and is not relevant to the choice of reflections for refinement. *R*-factors based on *F*^2^ are statistically about twice as large as those based on *F*, and *R*-factors based on ALL data will be even larger.

### Fractional atomic coordinates and isotropic or equivalent isotropic displacement parameters (Å^2^)

**Table:**

	*x*	*y*	*z*	*U*_iso_*/*U*_eq_	
Co1	1.0000	0.0000	0.0000	0.02745 (10)	
O1	0.78213 (19)	0.21130 (18)	0.03439 (4)	0.0354 (3)	
O2	1.0428 (2)	0.45515 (17)	0.06128 (4)	0.0349 (3)	
O3	0.9861 (3)	0.1663 (2)	0.13936 (6)	0.0656 (4)	
O4	1.2715 (3)	0.2897 (3)	0.18485 (7)	0.0860 (6)	
O5	0.7121 (2)	−0.21593 (19)	−0.00252 (5)	0.0367 (3)	
H5A	0.572 (4)	−0.193 (3)	−0.0126 (7)	0.044*	
H5B	0.761 (4)	−0.309 (3)	−0.0197 (7)	0.044*	
O6	1.1243 (2)	−0.1312 (2)	0.06473 (5)	0.0396 (3)	
H6A	1.089 (4)	−0.249 (4)	0.0668 (7)	0.047*	
H6B	1.076 (4)	−0.079 (4)	0.0883 (8)	0.047*	
N1	1.0723 (3)	0.3042 (2)	0.16413 (5)	0.0449 (4)	
C1	0.8429 (3)	0.3604 (2)	0.06134 (5)	0.0302 (3)	
C2	0.6512 (3)	0.4255 (4)	0.09495 (7)	0.0465 (5)	
H2A	0.5280	0.5076	0.0776	0.056*	
H2B	0.5702	0.3010	0.1056	0.056*	
C3	0.7378 (3)	0.5481 (3)	0.13750 (6)	0.0360 (4)	
C4	0.6146 (4)	0.7320 (3)	0.14716 (7)	0.0477 (5)	
H4	0.4881	0.7762	0.1265	0.057*	
C5	0.6733 (4)	0.8505 (3)	0.18614 (7)	0.0501 (5)	
H5	0.5823	0.9693	0.1920	0.060*	
C6	0.8658 (4)	0.7945 (3)	0.21649 (7)	0.0478 (5)	
H6	0.9062	0.8753	0.2427	0.057*	
C7	0.9977 (3)	0.6180 (3)	0.20765 (6)	0.0419 (4)	
H7	1.1312	0.5804	0.2274	0.050*	
C8	0.9304 (3)	0.4964 (2)	0.16911 (6)	0.0340 (4)	

### Atomic displacement parameters (Å^2^)

**Table:**

	*U*^11^	*U*^22^	*U*^33^	*U*^12^	*U*^13^	*U*^23^
Co1	0.01995 (16)	0.02718 (16)	0.03467 (18)	0.00213 (11)	−0.00444 (11)	−0.00896 (12)
O1	0.0242 (5)	0.0371 (6)	0.0445 (7)	0.0033 (5)	−0.0030 (5)	−0.0170 (5)
O2	0.0296 (6)	0.0312 (6)	0.0437 (7)	0.0001 (5)	−0.0005 (5)	−0.0103 (5)
O3	0.0995 (13)	0.0358 (8)	0.0613 (9)	0.0115 (8)	0.0019 (9)	−0.0049 (7)
O4	0.0700 (11)	0.0865 (13)	0.0987 (14)	0.0409 (10)	−0.0245 (10)	−0.0129 (11)
O5	0.0227 (6)	0.0368 (6)	0.0501 (7)	0.0005 (5)	−0.0034 (5)	−0.0142 (6)
O6	0.0452 (7)	0.0307 (6)	0.0417 (7)	0.0038 (5)	−0.0094 (6)	−0.0072 (5)
N1	0.0545 (10)	0.0427 (9)	0.0380 (8)	0.0124 (7)	0.0064 (7)	0.0062 (7)
C1	0.0244 (8)	0.0328 (8)	0.0328 (8)	0.0075 (6)	−0.0058 (6)	−0.0059 (6)
C2	0.0287 (9)	0.0635 (12)	0.0468 (11)	0.0016 (8)	−0.0009 (8)	−0.0240 (9)
C3	0.0286 (9)	0.0451 (9)	0.0345 (9)	0.0011 (7)	0.0027 (7)	−0.0089 (7)
C4	0.0394 (10)	0.0592 (12)	0.0439 (10)	0.0161 (9)	−0.0047 (8)	−0.0120 (9)
C5	0.0569 (12)	0.0461 (11)	0.0473 (11)	0.0134 (9)	0.0030 (9)	−0.0123 (9)
C6	0.0560 (12)	0.0498 (11)	0.0374 (10)	0.0006 (9)	0.0010 (8)	−0.0146 (8)
C7	0.0417 (10)	0.0530 (11)	0.0305 (8)	0.0021 (8)	−0.0034 (7)	−0.0011 (8)
C8	0.0351 (9)	0.0353 (9)	0.0319 (8)	0.0026 (7)	0.0054 (7)	0.0003 (7)

### Geometric parameters (Å, º)

**Table:**

Co1—O1^i^	2.0810 (11)	C1—C2	1.511 (2)
Co1—O1	2.0810 (11)	C2—C3	1.505 (2)
Co1—O5	2.0925 (12)	C2—H2A	0.9700
Co1—O5^i^	2.0925 (12)	C2—H2B	0.9700
Co1—O6	2.1141 (13)	C3—C8	1.391 (2)
Co1—O6^i^	2.1141 (13)	C3—C4	1.394 (3)
O1—C1	1.2637 (18)	C4—C5	1.374 (3)
O2—C1	1.2473 (19)	C4—H4	0.9300
O3—N1	1.214 (2)	C5—C6	1.376 (3)
O4—N1	1.213 (2)	C5—H5	0.9300
O5—H5A	0.82 (2)	C6—C7	1.373 (3)
O5—H5B	0.82 (2)	C6—H6	0.9300
O6—H6A	0.78 (2)	C7—C8	1.385 (2)
O6—H6B	0.81 (2)	C7—H7	0.9300
N1—C8	1.468 (2)		
			
O1^i^—Co1—O1	180.0	O2—C1—C2	119.65 (14)
O1^i^—Co1—O5	89.61 (5)	O1—C1—C2	115.40 (14)
O1—Co1—O5	90.39 (5)	C3—C2—C1	117.33 (14)
O1^i^—Co1—O5^i^	90.39 (5)	C3—C2—H2A	108.0
O1—Co1—O5^i^	89.61 (5)	C1—C2—H2A	108.0
O5—Co1—O5^i^	180.0	C3—C2—H2B	108.0
O1^i^—Co1—O6	89.23 (5)	C1—C2—H2B	108.0
O1—Co1—O6	90.77 (5)	H2A—C2—H2B	107.2
O5—Co1—O6	88.44 (5)	C8—C3—C4	115.41 (16)
O5^i^—Co1—O6	91.56 (5)	C8—C3—C2	126.60 (16)
O1^i^—Co1—O6^i^	90.77 (5)	C4—C3—C2	117.98 (16)
O1—Co1—O6^i^	89.23 (5)	C5—C4—C3	122.40 (18)
O5—Co1—O6^i^	91.56 (5)	C5—C4—H4	118.8
O5^i^—Co1—O6^i^	88.44 (5)	C3—C4—H4	118.8
O6—Co1—O6^i^	180.00 (6)	C4—C5—C6	120.38 (18)
C1—O1—Co1	130.14 (10)	C4—C5—H5	119.8
Co1—O5—H5A	125.5 (15)	C6—C5—H5	119.8
Co1—O5—H5B	103.7 (14)	C7—C6—C5	119.33 (17)
H5A—O5—H5B	104 (2)	C7—C6—H6	120.3
Co1—O6—H6A	112.4 (16)	C5—C6—H6	120.3
Co1—O6—H6B	117.5 (16)	C6—C7—C8	119.51 (17)
H6A—O6—H6B	104 (2)	C6—C7—H7	120.2
O4—N1—O3	122.64 (18)	C8—C7—H7	120.2
O4—N1—C8	118.61 (17)	C7—C8—C3	122.90 (16)
O3—N1—C8	118.75 (16)	C7—C8—N1	115.63 (16)
O2—C1—O1	124.94 (15)	C3—C8—N1	121.46 (15)
			
Co1—O1—C1—O2	25.0 (2)	C6—C7—C8—C3	2.2 (3)
Co1—O1—C1—C2	−156.08 (13)	C6—C7—C8—N1	−176.35 (17)
O2—C1—C2—C3	−20.9 (3)	C4—C3—C8—C7	−0.1 (3)
O1—C1—C2—C3	160.19 (17)	C2—C3—C8—C7	−179.68 (18)
C1—C2—C3—C8	−50.9 (3)	C4—C3—C8—N1	178.29 (16)
C1—C2—C3—C4	129.5 (2)	C2—C3—C8—N1	−1.3 (3)
C8—C3—C4—C5	−2.3 (3)	O4—N1—C8—C7	−19.3 (3)
C2—C3—C4—C5	177.3 (2)	O3—N1—C8—C7	160.92 (17)
C3—C4—C5—C6	2.6 (3)	O4—N1—C8—C3	162.21 (19)
C4—C5—C6—C7	−0.5 (3)	O3—N1—C8—C3	−17.6 (3)
C5—C6—C7—C8	−1.8 (3)		

Symmetry code: (i) −*x*+2, −*y*, −*z*.

### Hydrogen-bond geometry (Å, º)

**Table:**

*D*—H···*A*	*D*—H	H···*A*	*D*···*A*	*D*—H···*A*
O5—H5*A*···O1^ii^	0.82 (2)	2.00 (2)	2.7984 (16)	166 (2)
O5—H5*B*···O2^i^	0.82 (2)	1.89 (2)	2.6797 (17)	161 (2)
O6—H6*A*···O2^iii^	0.78 (2)	1.93 (2)	2.6979 (17)	169 (2)
O6—H6*B*···O3	0.81 (2)	2.22 (2)	2.988 (2)	159 (2)

Symmetry codes: (i) −*x*+2, −*y*, −*z*; (ii) −*x*+1, −*y*, −*z*; (iii) *x*, *y*−1, *z*.
