# Supplementary figures and images for: Tetra­aqua­bis­[2-(2-nitro­phen­yl)acetato-κO]cobalt(II)
Source: Acta Crystallogr E Crystallogr Commun. 2015 Feb 11;71(Pt 3):m59–60. doi: 10.1107/S2056989015002467 (PMC4350698; doi:10.1107/S2056989015002467)

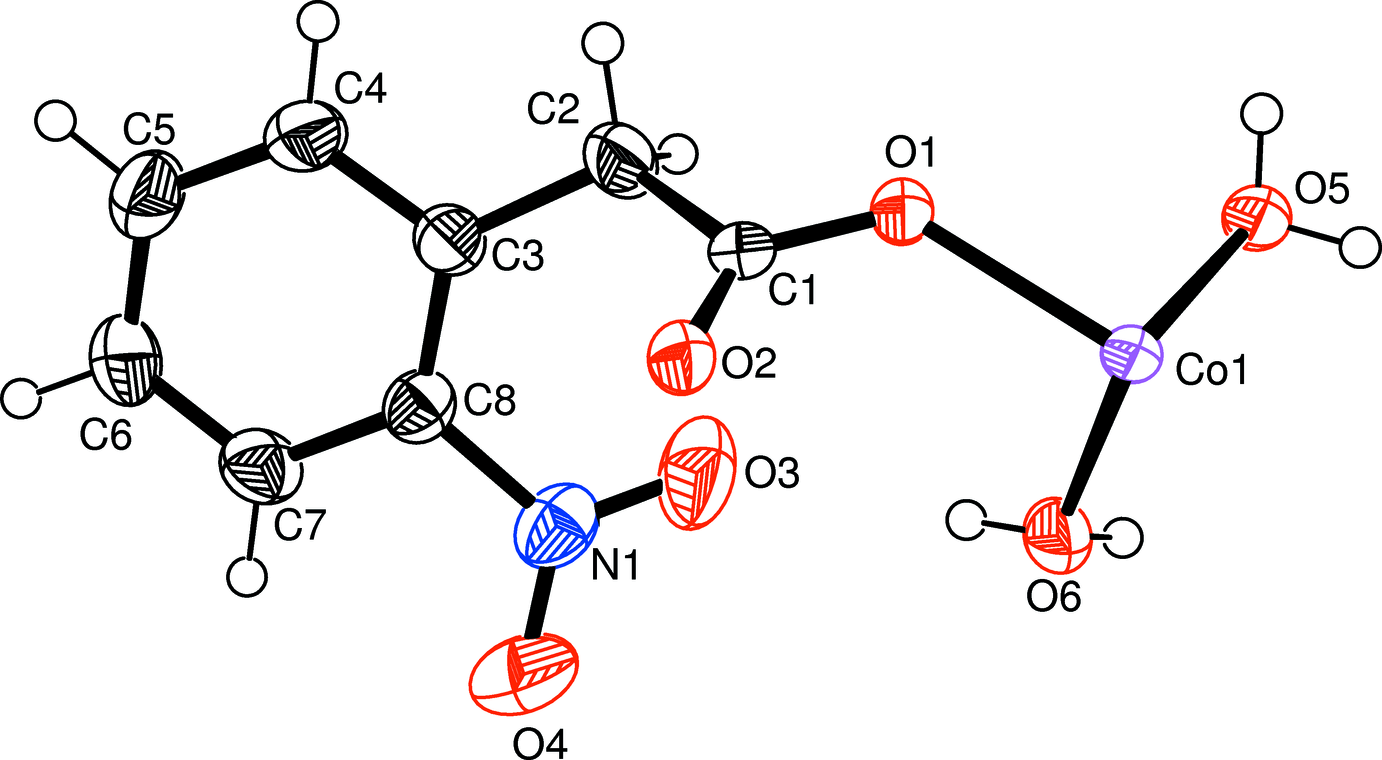

Supplement: Supplementary file 3 [file e-71-00m59-fig1.tif]

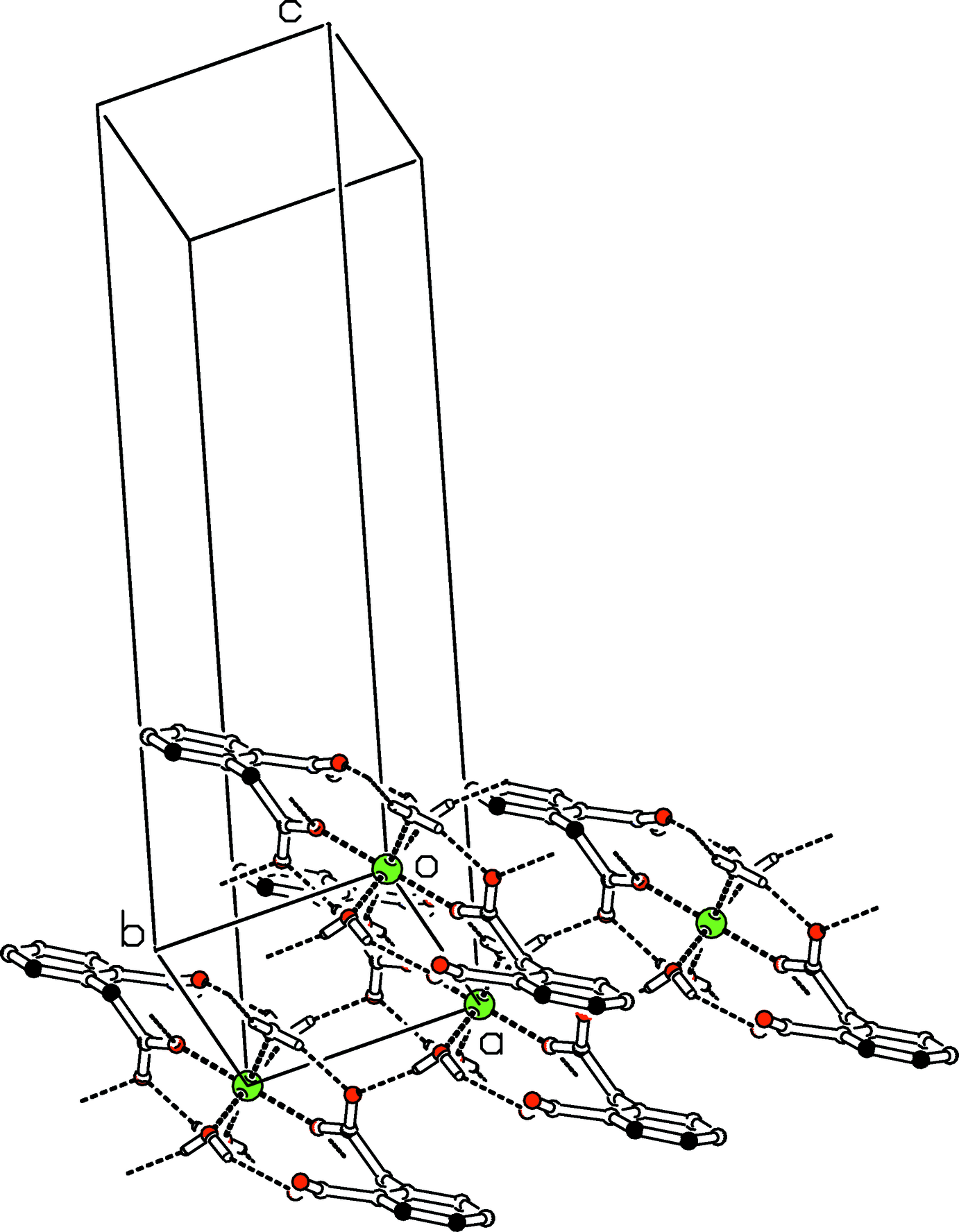

Supplement: Supplementary file 4 [file e-71-00m59-fig2.tif]
